# Translating human information into robot tasks: action sequence recognition and robot control based on human motions

**DOI:** 10.3389/frobt.2025.1462833

**Published:** 2025-06-23

**Authors:** Taichi Obinata, Kazutomo Baba, Akira Uehara, Hiroaki Kawamoto, Yoshiyuki Sankai

**Affiliations:** ^1^ Graduate School of Science and Technology, University of Tsukuba, Tsukuba, Japan; ^2^ CYBERDYNE, Inc., Tsukuba, Japan; ^3^ Center for Cybernics Research, University of Tsukuba, Tsukuba, Japan; ^4^ Institute of Systems and Information Engineering, University of Tsukuba, Tsukuba, Japan

**Keywords:** cybernics, action sequence recognition, long-horizontal task execution, 3D human skeletal information utilization, human robot interaction

## Abstract

Long-term use and highly reliable batteries are essential for wearable cyborgs including Hybrid Assistive Limb and wearable vital sensing devices. Consequently, there is ongoing research and development aimed at creating safer next-generation batteries. Researchers, leveraging advanced specialized knowledge and skills, bring products to completion through trial-and-error processes that involve modifying materials, shapes, work protocols, and procedures. When robots can undertake the tedious, repetitive, and attention-demanding tasks currently performed by researchers within facility environments, it will reduce the workload on researchers and ensure reproducibility. In this study, aiming to reduce the workload on researchers and ensure reproducibility in trial-and-error tasks, we proposed and developed a system that collects human motion data, recognizes action sequences, and transfers both physical information (including skeletal coordinates) and task information to a robot. This enables the robot to perform sequential tasks that are traditionally performed by humans. The proposed system employs a non-contact method to acquire three-dimensional skeletal information over time, allowing for quantitative analysis without interfering with sequential tasks. In addition, we developed an action sequence recognition model based on skeletal information and object detection results, which operated independent of background information. This model can adapt to changes in work processes and environments. By translating the human information including the physical and semantic information of a sequential task performed by humans into a robot, the robot can perform the same task. An experiment was conducted to verify this capability using the proposed system. The proposed action sequence recognition method demonstrated high accuracy in recognizing human-performed tasks with an average Edit score of 95.39 and an average F1@10 score of 0.951. In two out of the four trials, the robot adapted to changes in work processes without misrecognizing action sequences and seamlessly executed the sequential task performed by the human. In conclusion, we confirmed the feasibility of using the proposed system.

## 1 Introduction

Japan has become a super-aging society ahead of the rest of the world and is facing problems such as increasing medical costs, labor shortages, and a shortage of physicians in rural areas (Muramatsu and Akiyama, 2011; Cabinet Office Japan, 2024). To address these problems, it is important to extend healthy life expectancy, provide preventive healthcare, and offer telemedicine. For example, the lower limb, lumbar, and single joint types of Hybrid Assistive Limb can improve, support, enhance, and regenerate the wearer’s physical functions according to their intentions. These wearable cyborgs are widely used for treating neuromuscular diseases and improving motor functions in the elderly. Healthcare data collected by wearable vital sensing devices monitor the health status of individuals and are expected to be used by doctors for diagnosis and treatment. Ensuring a safe and efficient power supply is crucial for wearable cyborgs and wearable vital sensing devices worn for long-term use and extended periods of time. Apart from commonly used lithium-ion batteries, ongoing research and development are focused on next-generation batteries incorporating graphene to achieve higher safety standards (Cheng et al., 2011). Researchers with specialized knowledge and skills conduct the assembly of products through a trial-and-error process by varying the materials, shapes, work content, and procedures. The development phase involves simple repetitive tasks necessary for producing several to several dozen products required for product testing or demonstration. However, these tasks increase the workload of researchers and reduce the time available for intellectual tasks. They also pose a challenge in ensuring the reproducibility of quality due to human involvement (Shimizu et al., 2020; Burger et al., 2020).

**FIGURE 1:**
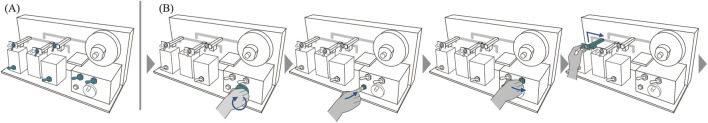
Examples of tasks in next-generation battery research and development: **(A)** Multiple toggle switches, each serving a different function. **(B)** Task requiring to be performed at the precise time, place, and sequence.

Using robots to handle the tedious, repetitive, and concentration-intensive tasks traditionally performed by researchers could substantially reduce the workload of these researchers and enhance reproducibility, leveraging existing facilities. However, achieving effective task delegation to robots necessitates developing methods capable of accommodating changes in task requirements resulting from trial-and-error in human work environment, while managing tasks across multiple processes. Moreover, completion of tasks in this study requires precise positioning and adherence to specific sequences (Figure 1). Furthermore, the environment contains multiple objects with identical names, making it impractical to perform tasks by selecting any one of them.

Conventional methods for teaching robots tasks include online teaching, where a person with specialized skills instructs a robot while operating it, and direct teaching, wherein a person physically manipulates the robot to demonstrate tasks (Khatib and Siciliano, 2016). These methods require each step of a task to be explicitly taught to the robot, and this process must be repeated whenever there are changes in the work process or environment. This makes it difficult to respond flexibly. In imitation learning, agents learn to replicate the actions of experts using demonstration and teleoperation data. Frameworks such as Mobile ALOHA, Play-LMP, and MimicPlay have been proposed to enable robots to handle tasks that involve multiple subtasks (Fu et al., 2024; Lynch et al., 2020; Wang et al., 2023). These frameworks aim to achieve generalization performance for latent plans, facilitating unseen transitions between subtasks. Given a target state, it is possible to generate actions (latent plans) that accomplish necessary subtasks to transition from the current state to the target state. However, in MimicPlay, a previous study, the success rate for tasks involving three or more subtasks remains at approximately 70%. Handling transitions between subtasks that are not included in the training data is challenging, and it is impractical to prepare training datasets that comprehensively cover all task variations arising from trial and error. A multimodal robot task planning pipeline utilizing GPT-4V, a vision-language model (VLM) trained on large datasets, has highlighted limitations in understanding videos (Wake et al., 2024). They were able to correctly transcribe task instructions from human-performed task videos in only 20.7% of cases. Moreover, to generate executable commands for robots, input text (prompts) must be provided to the VLM. The model’s output heavily depends on how the prompt is formulated, and since its internal structure is a black box, there is no clear optimal way to design prompts. As a result, achieving a high success rate for specific tasks remains challenging. Methods for generating commands from videos outline task sequences but do not account for spatial locations (Anh et al., 2018; Yang et al., 2023). Merely conveying the names of actions via text or voice proves insufficient. For these reasons, conventional approaches such as imitation learning and command generation from videos have difficulty adapting to task changes and enabling robots to share sequential tasks with a high success rate.

Despite advancements in robot manipulation capabilities for various tasks, achieving tasks that require execution at the appropriate position and in the correct sequence, while adapting to changing procedures, necessitates analyzing human tasks, acquiring spatial information about the performed actions, and identifying each action within a task, as demonstrated by humans to others. Furthermore, developing a system for translating this information to robots is essential. Our previous research focuses on a non-contact measurement system for 3D skeletal coordinates for the entire body, including fingers, and a method for simultaneously recognizing full-body and hand actions (Obinata et al., 2020; Obinata et al., 2023). By measuring, analyzing, and leveraging this human information, we believe robots can adapt to changes in work processes and environments based on a single human demonstration, even without specialized robot teaching expertise.

In this study, aiming to reduce the workload on researchers and ensure reproducibility in trial-and-error tasks, we proposed and developed a system that collects human motion data, recognizes action sequences, and translates both physical information (including skeletal coordinates) and the details of the task into a robot task. This enables the robot to execute sequential tasks demonstrated by humans. Furthermore, through a basic experiment applying the proposed system to a human task and having the robot perform the task in a simulated workplace, we confirmed the feasibility of the proposed system.

## 2 Methodology

**FIGURE 2:**
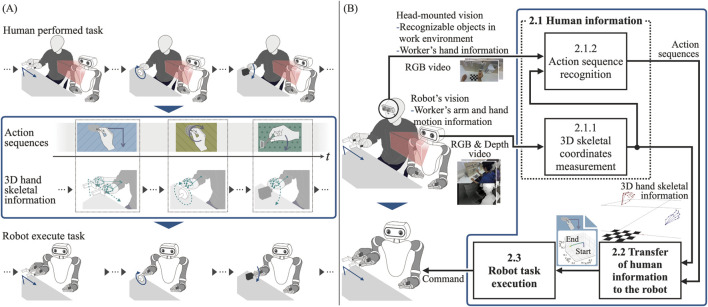
**(A)** Overview of the proposed system. **(B)** Detailed structural representation illustrating the major components and processing flow.

Figure 2A provides an overview of the proposed system, aimed at analyzing human-performed task and translating the human information, including physical and semantic aspects, of a sequential task into the robot. The proposed system acquires three-dimensional (3D) skeletal information as motion data from videos of humans performing tasks. Moreover, it identifies the action sequences involved in these tasks. Subsequently, based on the physical and semantic information from this analysis, the actions that constitute the task and the corresponding 3D skeletal information are communicated to the robot. The robot then executes these actions and performs the task in the same manner as a human would. Figure 2B illustrates the detailed structure of the proposed system, including its major components. The functionality of each block will be explained in the following sections.

### 2.1 Human information

#### 2.1.1 3D skeletal coordinates measurement

As part of our analysis of human motion, we measured time-series 3D skeletal coordinates using a noncontact method that does not interfere with tasks. We recorded videos containing both Red-Green-Blue (RGB) images and depth data using an RGB-Depth (RGB-D) camera. By applying 3D hand pose estimation to consecutive RGB frames, we extracted the time-series 3D skeletal coordinates for both the left and right hands of the worker. For 3D hand pose estimation, we utilized a pretrained Mediapipe model (Zhang et al., 2020), which accurately and rapidly estimates the 3D coordinates of 21 major joints of the hand. The origin of these 3D skeletal coordinates is approximately at the center of the hand, making them unsuitable for capturing spatial movements. To accurately reflect hand movements, we adjusted the origin of the skeletal 3D information for each hand to transform the 3D coordinate values obtained using the RGB-D camera. Subsequently, the coordinate system of the skeletal information was transformed from the coordinate system of the camera to the work coordinate system defined by a calibration board installed in the work environment. The extrinsic camera parameters, necessary for this transformation and representing the camera position and orientation, were calculated using Zhang’s method (Zhang, 2000). This process enabled us to measure the 3D skeletal coordinates of the hands during the task.

#### 2.1.2 Action sequence recognition

There are several methods for inferring the start and end times of actions in videos, including temporal action segmentation and temporal action detection (Wang et al., 2024; Ding et al., 2024). The datasets commonly used to evaluate these methods include GTEA, 50 Salads, Breakfast, and Assembly101, which focus on cooking and assembly activities (Li et al., 2015; Stein and McKenna, 2013; Kuehne et al., 2014; Sener et al., 2022). However, since these datasets do not include the specific actions targeted in our study, we developed an action sequence recognition model using our own dataset.

Image-based approaches often utilize pre-trained models such as ResNet, I3D, which have been trained on large datasets, to obtain feature tensors for single frames or a sequence of frames within a certain window size. Subsequently, there have been attempts to achieve high recognition accuracy by learning the temporal relationships between actions using these feature tensors. However, even with these large datasets, it is challenging to obtain features with a universal spatial and temporal representation (Wang et al., 2024). Since image features change with background variations, it is difficult to develop models with sufficient recognition accuracy unless training data from various environments is available.

Additionally, recognizing both action information (probes) and object information is crucial for understanding the action sequence of the target task. Robust skeletal inference and object detection techniques capable of handling changes in the background information have emerged. In this study, we developed a method to recognize both motion and object information by utilizing skeletal data and object detection results that are independent of background variations.

There are two approaches for time-series motion recognition: one involves sequentially taking inputs by applying a sliding window to consecutive frames, and the other involves simultaneously inputting all frames. The latter method often aims for highly accurate recognition by leveraging repetitions and relationships among actions during a task. However, in tasks where the order of actions can change, this assumption may not hold, and the recognition accuracy may suffer. Therefore, we adopted a method that uses a sliding window to acquire input data for the action recognition model, thereby enabling the recognition of time-series action sequences through repeated action recognition.

##### 2.1.2.1 Target action

There are five types of target action: actions commonly performed to operate mechanical devices, such as “flip a switch,” “push a button,” and “turn a handle”; an action involving “move while grasping an object” (in this case, moving a stage along a rail); and an action classified as “others,” which does not fall into the aforementioned categories.

##### 2.1.2.2 Action recognition model based on motion information

In this study, we adopted a skeleton-based action recognition model that is minimally affected by environmental changes such as background and illumination and can geometrically respond to variations in a camera’s angle of view. The 3D skeletal coordinates, which serve as the model’s input, were obtained by measuring the time series of the 3D skeletal coordinates, as described in Section 2.1.1.

The 3D skeletal coordinates were represented as static undirected graphs, with nodes (vertices) being the representative points of the skeleton, each holding 3D coordinate values. In this study, we used a Graph Convolutional Network (GCN), a type of neural network capable of handling data with a graph structure, as the action recognition model (Kipf and Welling, 2017). Specifically, we adopted the Spatial-temporal graph convolutional networks (ST-GCN), which efficiently aggregates information in both temporal and spatial dimensions (Yan et al., 2018).

The action recognition model based on motion information was pretrained using supervised learning on a custom dataset consisting of the movements of a single participant. The number of epochs was set to 50 and the batch size was 32. We employed the cross-entropy loss function, and optimization was performed using stochastic gradient descent with an initial learning rate of 0.1. The learning rate decreased as the training progressed: it was set to 0.1 for the first 15 epochs, 0.01 for the next 15 epochs, and 0.001 for the remaining epochs.

##### 2.1.2.3 Acquisition of target object information

To obtain object information in the vicinity of the hand, object detection and hand-pose estimation were performed on first-person videos. Yolo v8 was used for object detection, and Mediapipe was employed for hand pose estimation (Zhang et al., 2020). Using the coordinates of the fingertips and the distance between these coordinates and the center of the bounding box obtained from object detection, the closest object below a certain threshold distance was identified as the object near the finger. If no object was within the threshold distance, it was recorded as “Nothing.” The nearest object was identified for both the thumb and index finger. The object detection results were converted into 768-dimensional feature vectors using CLIP’s text encoder (Radford et al., 2021). This approach allows for the acquisition of information on objects near the fingers, independent of background information.

##### 2.1.2.4 Integration of the features of motion and object information

**FIGURE 3:**
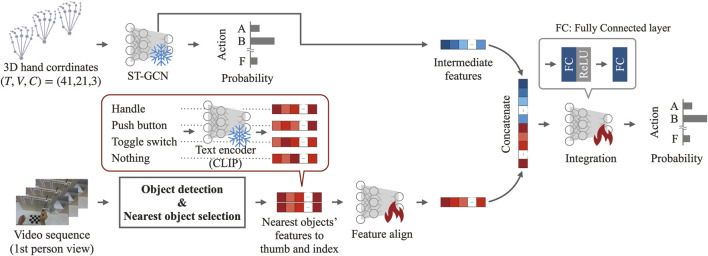
Model structure outline integrating motion and object information.

The features related to the motion information were the 128-dimensional intermediate output features of the model described in Section 2.1.2.2. The 768-dimensional features for each finger, acquired from the target object information, were mixed using a 1 × 1 convolution, followed by batch normalization and one-dimensional convolution. The object information was then transformed into 128 dimensions, matching the feature dimensions of the motion information, through fully connected (FC) layers. The features of the motion and object information were concatenated and passed through an FC layer, an activation function (ReLU), and another FC layer to output a one-hot encoding representing the class. An overview of the model structure that integrates motion and object information is shown in Figure 3.

Training of the model integrating features of motion and object information was performed using supervised learning with an original dataset consisting of actions from a single participant. The number of epochs was set to 50 and the batch size to 32. A cross-entropy loss function was used, with a combined weighted average loss function applied to the motion information, object information, and predicted labels after integration. The combined loss function assigned 75% weight to the loss from the predicted labels after integration, and 12.5% weight each to the losses from the motion and object information. Stochastic gradient descent with a learning rate of 0.01 was used as the optimization algorithm. The learning rate decreased sequentially with the number of epochs: 0.01 for up to 15 epochs, 0.001 for up to 30 epochs, and 0.0001 for the remaining epochs.

### 2.2 Transfer of human information to the robot

Based on the results of the action sequence recognition obtained from the task analysis in Section 2.1, the 3D skeletal coordinates (trajectory information) from the start to the end of each action were compiled and transferred to the robot. From the action labels for each frame obtained through action sequence recognition, we extracted intervals between “others” actions. Next, to mitigate the effects of over segmentation, we selected the most frequently occurring action labels within each interval. If the interval was smaller than half the window size of the action recognition model, it was excluded. The measured 3D skeletal coordinates included outliers from inference process failures, fluctuations in 3D hand pose estimation, and noise in the depth information from the RGB-D camera. We removed outliers by applying a Hampel filter with a window size of 15, and reduced noise by applying a simple moving average filter with a window size of 3 to smooth the data.

### 2.3 Robot task execution

#### 2.3.1 Robot execution of each action

Based on the action sequence recognition results and the 3D skeletal trajectory information from the start to the end of each action provided by the proposed system, the robot performed each action and completed the entire task. Initially, the end effector was moved to the 3D coordinates of the skeleton at the start of a given action. Subsequently, the robot reached the target object while detecting it using a camera mounted on the end effector. Yolo Tiny, a lightweight model with fast inference, was used for object detection (Bochkovskiy et al., 2020). The objects targeted by the detection model include toggle switches, push switches, and handles.

#### 2.3.2 Robot

**FIGURE 4:**
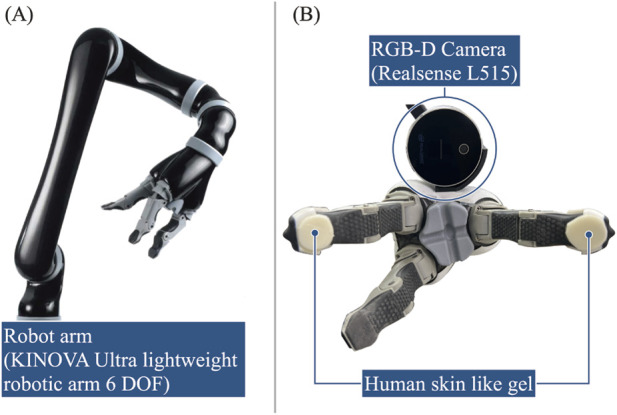
The robot employed: **(A)** Robotic arm [adapted from (Kinova inc., 2018)]. **(B)** Custom end effector.

In this study, we utilized the Kinova JACO2 robotic arm, specifically designed to assist individuals with physical disabilities in their daily activities, and to operate in environments where humans and robots interact. This robotic arm features six degrees of freedom and an end effector with three fingers. Additionally, as depicted in Figure 4, an RGB-D camera (Realsense L515, Intel Corporation) was mounted on the end effector to calibrate the robot’s position and orientation, as well as for object recognition. A gel-like resin with properties similar to those of the human skin was applied to the fingertips to compensate for positioning errors during object manipulation, alongside enhancing the gripping force.

#### 2.3.3 Calibrating the robot’s coordinate system

To make the measured time-series 3D skeleton information usable for controlling the robot arm, it is crucial to establish a homogeneous transformation matrix between the robot coordinate system and the work coordinate system. When the robot is installed in its environment, a homogeneous transformation matrix can be accurately obtained at the design stage or through direct measurements. However, when a robot arm is installed as needed, a calibration method is required to derive the appropriate homogeneous transformation matrix. Therefore, we calibrated the position and orientation of the robot using an RGB camera mounted on the end effector. The homogeneous transformation matrix between the robot and camera coordinate systems was calculated based on the joint angles of the robot arm. Once the homogeneous transformation matrix between the work and camera coordinate systems is determined, it can be multiplied by the transformation matrix between the robot and camera coordinate systems to obtain the transformation matrix between the robot and work coordinate systems. The homogeneous transformation matrix between the camera and work coordinate systems was calculated using Zhang’s method (Zhang, 2000).

## 3 Experiments

**FIGURE 5:**
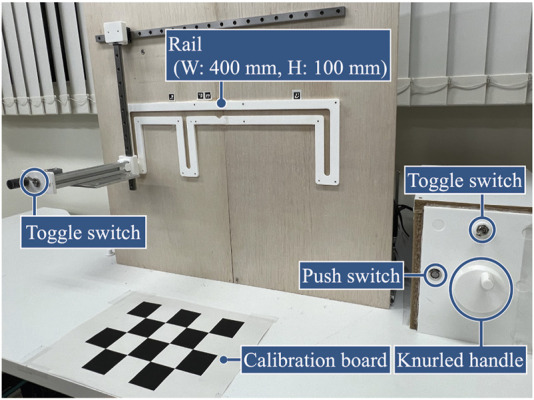
Simulated environment.

**FIGURE 6:**
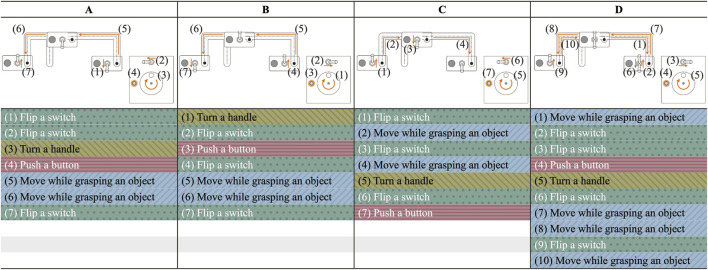
Action sequence for each task (Trials **(A–D)**), along with their positions in the simulated environment.

To confirm the feasibility of the proposed system, we conducted an experiment to verify that the robot can correctly recognize the names and sequences of each action that constitutes a human-performed task, and that the robot can perform the task using the action sequence recognition results and the 3D skeletal coordinates of the time series for control. The experimental environment simulated an actual work environment (Figure 5). The participants were three males aged 22–26 years, one of whom was used as training data for the action sequence recognition model (the person who performed Trial D). Consent was obtained from the participants before the experiment. The participants used their left and right hands to perform tasks in different sequences. Four different tasks (Trials A, B, C, and D) were performed. Figure 6 shows a graphical representation of the action sequence for each task along with their positions in the simulated environment.

We captured videos of a human performing a task using three RGB-D cameras (one installed at the top and two positioned behind the left and right sides). Using the proposed system, human information (such as action sequences and 3D skeletal coordinates) from the captured video was transferred to the robot. Subsequently, the robot executed the tasks accordingly. If errors were found in the recognition results, they were corrected to the correct action names, and any unnecessary actions were eliminated.

We calculated the Edit score, which indicates the closeness of the recognized action sequence to the correct action sequence, and F1@k, which evaluates the temporal segmentation quality of the action sequence (Lea et al., 2016; Lea et al., 2017). The success or failure of the robot task execution was evaluated based on whether the robot could perform each action and complete the entire task. The success conditions for each action are listed in Table 1. Furthermore, the feasibility of the proposed system was confirmed by its ability to seamlessly transition from recognizing action sequences performed by humans to executing the same tasks by robots.

**TABLE 1:** Success condition for each action.

Action	Success conditions
Move while grasping an object	Whether the handle was turned successfully
Flip a switch	Whether the toggle switch was operated successfully
Push a button	Whether the button was pressed successfully
Turn a handle	Whether the object was moved to the designated position successfully

## 4 Results

### 4.1 Accuracy in recognizing action sequence

**FIGURE 7:**
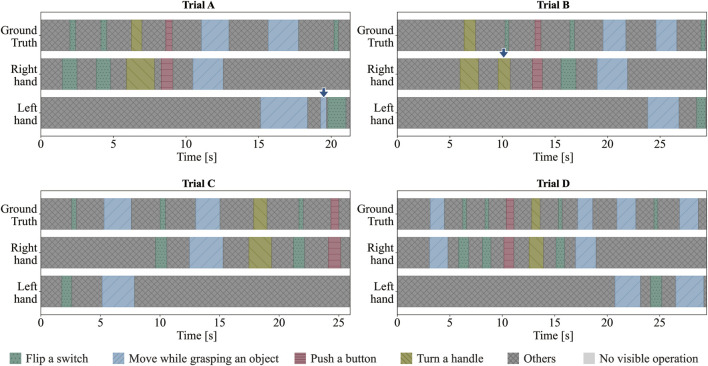
Results of action sequence recognition for each task [Trials **(A-D)**].

Figure 7 displays the results of the action sequence recognition for each trial. In Trial A, there was an unnecessary recognition of the action “Move while grasping an object,” and in Trial B, “Flip a switch” was misrecognized as “Turn a handle” (indicated by the blue arrows in Figure 7 for trials A and B, respectively). Table 2 lists the F1@{10, 25, 50} and the Edit scores for each trial.

**TABLE 2:** F1@{10, 25, 50} scores and the Edit score.

Trial	F1@10	F1@25	F1@50	Edit score
A	0.938	0.875	0.500	88.24
B	0.933	0.933	0.800	93.33
C	0.933	0.933	0.800	100.00
D	1.000	1.000	0.762	100.00
Average	0.951	0.935	0.716	95.39

### 4.2 Robot task execution

**FIGURE 8:**
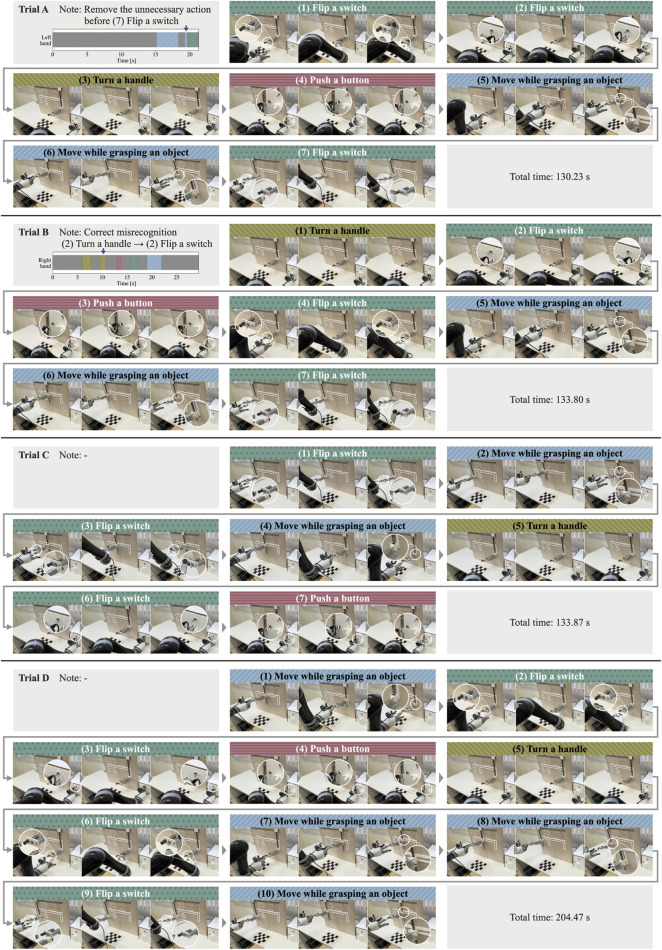
Results of robot task execution.

Figure 8 shows a robot executing tasks in chronological order. We confirmed that all the actions met the respective success criteria. In addition, the time required to complete each trial, from the first action to the last, was 130.23 s for Trial A, 133.80 s for Trial B, 133.87 s for Trial C, and 204.47 s for Trial D. The longer completion time for Trial D was attributed to the greater number of actions involved, as Trial D consisted of 10 actions, whereas Trials A, B, and C consisted of 7 actions each.

## 5 Discussions

### 5.1 Considerations of the proposed system

The average Edit score for all four trials was 95.39, indicating the high accuracy of the model in recognizing human-performed action sequences. The Edit score decreased due to unnecessary recognition in Trial A and misrecognition in Trial B. The unnecessary recognition in Trial A was caused by the participant opening their fingers widely while reaching the toggle switch, leading the system to incorrectly identify the neighboring object as a handle instead of the toggle switch. Improving the method for acquiring object information is expected to reduce unnecessary recognition. In Trial B, the action recognition results before post-processing showed correct recognition at the “Flip a switch” point, but there were misrecognitions of “Turn a handle” before and after that point. Consequently, the action transmitted to the robot was incorrectly labeled as “Turn a handle.” Increasing the training data and improving the recognition accuracy of the action recognition model are expected to achieve more accurate recognition of action sequences. The average F1@10 score was 0.951, whereas the average F1@50 score, which was the most stringent measure, was 0.716. “Flip a switch” was the most common action that resulted in a decrease in the F1@50 score. This action is shorter in duration compared to other actions, making it difficult to distinguish from “other” actions before and after it during the annotation of the training data. Improvements, such as reviewing the training data annotations and changing the input data for the action recognition model to the differences in skeletal information between frames, are expected to improve temporal recognition accuracy and reduce misrecognition.

Trial D was performed by an individual whose data were used to train the action-recognition model. The Edit score was 100, and the F1@10 and F1@25 scores were 1.00, indicating that the action sequence was accurately recognized over the time axis. Furthermore, trials A, B, and C, conducted by participants not included in the training data, achieved similarly high recognition accuracies with F1@10 scores of 0.938, 0.933, and 0.933, respectively, with the edit score of 100 in trial C. This demonstrates that the action recognition model trained on the actions of a specific individual can also accurately recognize the action sequences of the tasks of other individuals. We considered the developed action sequence recognition model to be sufficient to recognize the target task. Additionally, these results suggest the potential to reduce the cost of collecting learning data in real-world settings and to enable the recognition of other researchers’ action sequence based on the data from a single researcher. This capability may be attributed to our approach of integrating motion and object information. Unlike conventional image- and video-based methods, this approach reduces the learning burden and enables explicit action understanding. The results of action sequence recognition experiments confirm that action sequence recognition is feasible through the integration of motion and object information.

From the perspective of the success rate of tasks performed by robots, end-to-end methods, including reinforcement learning and imitation learning from environmental recognition to control, have become capable of dealing with unseen environments in manipulation tasks. However, even with large-scale datasets and large networks, such as Open-X, the success rate remains relatively low at approximately 62% (Maddukuri et al., 2023). In all four trials (31 actions), the robot was able to move the end effector to the position of the 3D skeletal coordinates at the start time given by the proposed system and perform the corresponding actions, successfully completing the task in each trial. This indicated that the control of the implemented robot was sufficient for the target task. In the implemented robot control, the time required for the robot to complete the tasks was longer than that of a human. In this experiment, task execution accuracy was prioritized as a critical evaluation metric. Consequently, software limiters on movement speed for safety and the accumulation of waiting time to enhance object detection reliability resulted in longer task execution times compared to humans. Nevertheless, all actions successfully met the predefined success criteria, demonstrating the robot’s ability to perform tasks with a comparable level of accuracy to humans. While this highlights the current challenges in achieving both high accuracy and efficiency, future research should focus on improving task execution efficiency by optimizing control algorithms and enhancing hardware capabilities. In action sequence recognition, the action “move while grasping an object” is treated as a single label, but in reality, it encompasses movements in different directions. By simultaneously acquiring skeletal information while analyzing a human task, the action label can be transferred to the robot without distinguishing between all the movement patterns in action sequence recognition. Furthermore, by using the trajectory of the acquired skeletal information for control, the robot can respond to movements in different directions without having to prepare the control for all movement patterns.

In Trials C and D, the robot successfully completed the tasks without any modifications to the action sequence recognition. This confirms the feasibility of the proposed system. Due to errors in the recognition results of the action sequence in Trials A and B, it was not possible to seamlessly transfer the task information performed by the human to the robot. However, in both Trials A and B, there were no missed actions due to errors in the recognition results; instead, the errors involved incorrect action names and the recognition of extra sequences. It is easy to correct incorrect action names and delete unnecessary sequences by simply reviewing the video of the task. In addition, the robot could complete the task by making corrections and deletions, demonstrating the practicality of the proposed system for task analysis. In this study, misrecognitions in action sequence recognition were manually corrected; however, a self-correction mechanism for the robot is necessary. For example, an incorrect action may be detected when the robot moves its end-effector to the starting position of an action but finds that the object is missing. In such cases, the robot could revise its recognized action sequence by utilizing real-time information about the current state of the environment. Additionally, when the interval between two distinct actions is very short, humans can reasonably judge that it is unlikely to represent two separate actions based on common sense, allowing them to revise it as a single continuous action. To address these challenges, not only improving the accuracy of action sequence recognition but also incorporating post-processing mechanisms that enable the robot to utilize common-sense reasoning will be necessary in the future topic.

The purpose of this study is to confirm that a robot can perform sequential tasks for frequently changing work procedures in the research and development process based on human demonstrations. In this study, we evaluated the recognition accuracy of action sequences through a basic experiment in a controlled simulated environment and confirmed that the robot successfully executed the task. However, in real-world environments, background clutter may affect the performance of the proposed system. In this study, we use skeletal information for action recognition based on motion information, and in the integration of object and motion information, object information features are represented as linguistic embeddings using CLIP. As a result, the system is less susceptible to environmental variations. Nevertheless, the object detection module, which extracts object information, may be influenced by environmental factors. The framework of the proposed method itself is not inherently dependent on the environment. By incorporating a more robust object recognition method, the system can be adapted to different environments.

Using the proposed system, researchers can perform their tasks as usual without making specific changes, and the robot can perform sequential tasks. The proposed system reduces the effort required for coding and adjustments compared to the conventional teaching playback process, which involves repeated operation and positioning of the robot. Furthermore, as researchers repeat their daily tasks, the proposed system allows the accumulation of data for the robot’s imitation learning.

### 5.2 Future works

In this study, we achieved high recognition accuracy even with a model trained on data from a single participant. In the future, we plan to incorporate motion data from participants with diverse physiques and movement styles to further improve recognition accuracy and robustness. The action sequence recognition of the proposed system was designed to achieve consistent accuracy, even when the background information varied, although we have not yet evaluated the system under these conditions. In the future, we plan to validate the system in actual production environments to confirm that it maintains high recognition accuracy in different settings. In the robot control of the developed system, execution is currently limited to feedforward control based on pre-defined motion patterns. As a result, for real-world deployment, it is necessary to integrate mechanisms for adaptation to environmental and situational changes, as well as learning capabilities, into the robot’s edge device. Brain-inspired motion control approaches offer the potential to achieve such adaptability through learning while maintaining low power consumption and real-time performance (Yang et al., 2022). By incorporating these models into the robot’s edge system, stable task success rates can be expected even under varying environmental and situational conditions. Additionally, the developed action sequence recognition method requires continuous operation of the recognition module, even during static periods. However, in real-world applications, this results in unnecessary computations on low-information segments unrelated to the target actions. This leads to inefficiencies in computational and energy resources—particularly when processing is confined to edge devices. In this context, approaches based on spiking neural networks (SNNs) offer promising potential, as their event-driven processing architecture performs computations only in response to input, thereby avoiding redundant processing (Yang et al., 2024; Yang and Chen, 2023b; Yang and Chen, 2023a). The adoption of SNNs for action sequence recognition may thus represent as an effective approach toward constructing practical and energy-efficient systems from the perspective of Embodied AI. In addition, we plan to evaluate the usability and acceptability of the proposed system. Furthermore, the proposed system could sequentially recognize tasks performed by humans and quantify their techniques as skeletal movements. This capability is expected to be useful in fields that depend on manual human skills such as life science experiments and cell manufacturing. We also plan to verify the applicability of the proposed system to these and other areas.

## 6 Conclusion

In this study, we aimed to reduce the workload on researchers and ensure reproducibility in trial-and-error tasks by proposing and developing a system that enables robots to perform sequential tasks executed by humans by collecting 3D skeletal information of human movements, recognizing action sequences, and translating both physical information (including skeletal coordinates) and the details of the task into the robot task. We conducted a basic experiment where we applied the proposed system to a human task and had the robot replicate it. The proposed system accurately recognized the action sequences of the human-performed task. In two out of four trials, the robot successfully and seamlessly replicated the human tasks. These results confirmed the feasibility of the proposed system.

## Data Availability

The raw data supporting the conclusions of this article will be made available by the authors, without undue reservation.

## References

[B1] Anh N. Kanoulas D. Muratore L. Caldwell D. G. Tsagarakis N. G. (2018). Translating videos to commands for robotic manipulation with deep recurrent neural networks. IEEE, 3782–3788.

[B2] Bochkovskiy A. Chien-Yao W. Hong-Yuan M. L. (2020). YOLOv4: optimal speed and accuracy of object detection. Ithaca: Cornell University Library.

[B3] Burger B. Maffettone P. M. Gusev V. V. Aitchison C. M. Bai Y. Wang X. (2020). A mobile robotic chemist. Nature 583 (7815), 237–241. 10.1038/s41586-020-2442-2 32641813

[B4] Cabinet Office Japan, (2024). Annual report on the ageing society [summary] FY2022.

[B5] Cheng Q. Tang J. Ma J. Zhang H. Shinya N. Qin L. (2011). Graphene and carbon nanotube composite electrodes for supercapacitors with ultra-high energy density. Phys. Chem. Chem. Phys. 13 (39), 17615–17624. 10.1039/c1cp21910c 21887427

[B6] Ding G. Sener F. Yao A. (2024). Temporal action segmentation: an analysis of modern techniques. IEEE Trans. pattern analysis Mach. Intell. 46 (2), 1011–1030. 10.1109/tpami.2023.3327284 37874699

[B7] Fu Z. Zhao T. Z. Finn C. (2024). Mobile aloha: learning bimanual mobile manipulation with low-cost whole-body teleoperation. arXiv Prepr. arXiv:2401.02117. 10.48550/arXiv.2401.02117

[B8] Khatib O. Siciliano B., (2016). Springer handbook of robotics. 2nd Edition. edn. Cham: Springer International Publishing.

[B9] Kipf T. N. Welling M. (2017). Semi-supervised classification with graph convolutional networks. Ithaca: Cornell University Library.

[B34] Kinova inc. (2018). Kinova Ultra lightweight robotic arm 6. DOF Specifications.

[B10] Kuehne H. Arslan A. Serre T. (2014). “The language of actions: recovering the syntax and semantics of goal-directed human activities,” in Proceedings of the IEEE conference on computer vision and pattern recognition 2014, 780–787. 10.1109/cvpr.2014.105

[B11] Lea C. Flynn M. D. Vidal R. Reiter A. Hager G. D. (2017). “Temporal convolutional networks for action segmentation and detection,” in proceedings of the IEEE Conference on Computer Vision and Pattern Recognition 2017, 156–165.

[B12] Lea C. Vidal R. Hager G. D. (2016). “Learning convolutional action primitives for fine-grained action recognition,” in 2016 IEEE international conference on robotics and automation (ICRA) 2016 (IEEE), 1642–1649.

[B13] Li Y. Ye Z. Rehg J. M. (2015). “Delving into egocentric actions,” in Proceedings of the IEEE conference on computer vision and pattern recognition 2015, 287–295. 10.1109/cvpr.2015.7298625 PMC4784702 26973427

[B14] Lynch C. Khansari M. Xiao T. Kumar V. Tompson J. Levine S. (2020). “Learning latent plans from play,” in Conference on robot learning 2020 (PMLR), 1113–1132. Available online at: https://proceedings.mlr.press/v100/lynch20a.html

[B15] Maddukuri A. Gupta A. Padalkar A. Lee A. Pooley A. Jain A. (2023). “Open X-embodiment: robotic learning datasets and RT-X models: open X-embodiment collaboration,” in 2024 IEEE International Conference on Robotics and Automation (ICRA) Oct 31, 2023, IEEE.

[B16] Muramatsu N. Akiyama H. (2011). Japan: super-aging society preparing for the future. Gerontologist 51 (4), 425–432. 10.1093/geront/gnr067 21804114

[B17] Obinata T. Kawamoto H. Sankai Y. (2023). [Study on Action Recognition Including Fine-detailed Hand Motion toward Monitoring System] Tearai sagyou no monitoring ni muketa zenshin to temoto no dousaninshiki syuhou no kenkyuu kaihatu. IPSJ J. Inf. Process. Soc. Jpn., 926–940. 10.20729/00225500

[B18] Obinata T. Kawamoto H. Sankai Y. (2020). Development of real-time assembly work monitoring system based on 3D skeletal model of arms and fingers, oct 11, 2020. IEEE, 363–368.

[B19] Radford A. Kim J. W. Hallacy C. Ramesh A. Goh G. Agarwal S. (2021). “Learning transferable visual models from natural language supervision,” in International conference on machine learning 2021 (PMLR), 8748–8763. Available online at: https://proceedings.mlr.press/v139/radford21a

[B20] Sener F. Chatterjee D. Shelepov D. He K. Singhania D. Wang R. (2022). “Assembly101: a large-scale multi-view video dataset for understanding procedural activities,” in Proceedings of the IEEE/CVF Conference on Computer Vision and Pattern Recognition 2022, 21096–21106.

[B21] Shimizu R. Kobayashi S. Watanabe Y. Ando Y. Hitosugi T. (2020). Autonomous materials synthesis by machine learning and robotics. Apl. Mater. 8 (11). 10.1063/5.0020370

[B22] Stein S. Mckenna S. J. (2013). Combining embedded accelerometers with computer vision for recognizing food preparation activities, 729–738.

[B23] Wake N. Kanehira A. Sasabuchi K. Takamatsu J. Ikeuchi K. (2024). GPT-4V(ision) for robotics: multimodal task planning from human demonstration. IEEE robotics automation Lett. 9 (11), 10567–10574. 10.1109/lra.2024.3477090

[B24] Wang B. Zhao Y. Yang L. Long T. Li X. (2024). Temporal action localization in the deep learning era: a survey. IEEE Trans. pattern analysis Mach. Intell. 46 (4), 2171–2190. 10.1109/tpami.2023.3330794 37930912

[B25] Wang C. Fan L. Sun J. Zhang R. Fei-Fei L. Xu D. (2023). “Mimicplay: long-horizon imitation learning by watching human play,” in 7th Conference on Robot Learning 2023, 201–221.

[B26] Yang S. Chen B. (2023a). Effective surrogate gradient learning with high-order information bottleneck for spike-based machine intelligence. IEEE transaction neural Netw. Learn. Syst. 36 (1), 1734–1748. 10.1109/tnnls.2023.3329525 37991917

[B27] Yang S. Chen B. (2023b). SNIB: improving spike-based machine learning using nonlinear information bottleneck. IEEE Trans. Syst. man, Cybern. Syst. 53 (12), 7852–7863. 10.1109/tsmc.2023.3300318

[B28] Yang S. He Q. Lu Y. Chen B. (2024). Maximum entropy intrinsic learning for spiking networks towards embodied neuromorphic vision. Neurocomputing 610, 128535. 10.1016/j.neucom.2024.128535

[B29] Yang S. Wang J. Zhang N. Deng B. Pang Y. Azghadi M. R. (2022). CerebelluMorphic: large-scale neuromorphic model and architecture for supervised motor learning. IEEE transaction neural Netw. Learn. Syst. 33 (9), 4398–4412. 10.1109/tnnls.2021.3057070 33621181

[B30] Yang S. Zhang W. Song R. Cheng J. Wang H. Li Y. (2023). Watch and act: learning robotic manipulation from visual demonstration. IEEE Trans. Syst. man, Cybern. Syst. 53 (7), 4404–4416. 10.1109/tsmc.2023.3248324

[B31] Yan S. Xiong Y. Lin D. (2018). “Spatial temporal graph convolutional networks for skeleton-based action recognition,” in Proceedings of the AAAI Conference on Artificial Intelligence, 32 (1). 10.1609/aaai.v32i1.12328

[B32] Zhang F. Bazarevsky V. Vakunov A. Tkachenka A. Sung G. Chang C. (2020). Mediapipe hands: on-device real-time hand tracking. arXiv Prepr. arXiv:2006.10214. 10.48550/arXiv.2006.10214

[B33] Zhang Z. (2000). A flexible new technique for camera calibration. IEEE Trans. pattern analysis Mach. Intell. 22 (11), 1330–1334. 10.1109/34.888718

